# Tumor suppressor FLCN inhibits tumorigenesis of a *FLCN-null *renal cancer cell line and regulates expression of key molecules in TGF-β signaling

**DOI:** 10.1186/1476-4598-9-160

**Published:** 2010-06-23

**Authors:** Seung-Beom Hong, HyoungBin Oh, Vladimir A Valera, Jaime Stull, Duy-Tan Ngo, Masaya Baba, Maria J Merino, W Marston Linehan, Laura S Schmidt

**Affiliations:** 1 Urologic Oncology Branch, Center for Cancer Research, National Cancer Institute, 10 Center Drive MSC1107, 10/CRC/1W-5940, Bethesda, MD 20892 USA; 2 Laboratory of Pathology, Center for Cancer Research, National Cancer Institute, 10 Center Drive MSC1516, 10/MCC/2B50, Bethesda, MD 20892 USA; 3 Basic Science Program, SAIC-Frederick, Inc, NCI-Frederick, Frederick, MD 21702 USA

## Abstract

**Background:**

Germline mutations in the *FLCN *gene are responsible for the development of fibrofolliculomas, lung cysts and renal neoplasia in Birt-Hogg-Dube' (BHD) syndrome. The encoded protein folliculin (FLCN) is conserved across species but contains no classic motifs or domains and its function remains unknown. Somatic mutations or loss of heterozygosity in the remaining wild type copy of the *FLCN *gene have been found in renal tumors from BHD patients suggesting that *FLCN *is a classic tumor suppressor gene.

**Results:**

To examine the tumor suppressor function of *FLCN*, wild-type or mutant *FLCN *(H255R) was stably expressed in a *FLCN-null *renal tumor cell line, UOK257, derived from a BHD patient. When these cells were injected into nude mice, tumor development was inversely dependent upon the level of wild-type *FLCN *expression. We identified genes that were differentially expressed in the cell lines with or without wild-type *FLCN*, many of which are involved in TGF-β signaling, including *TGF-β2 *(*TGFB2*)*, inhibin β A chain *(*INHBA*)*, thrombospondin 1 *(*THBS1*), *gremlin *(*GREM1*), and *SMAD3*. In support of the *in vitro *data, *TGFB2*, *INHBA*, *THBS1 *and *SMAD3 *expression levels were significantly lower in BHD-associated renal tumors compared with normal kidney tissue. Although receptor mediated SMAD phosphorylation was not affected, basal and maximal TGF-β-induced levels of *TGFB2*, *INHBA *and *SMAD7 *were dramatically reduced in *FLCN-null *cells compared with *FLCN*-restored cells. Secreted TGF-β2 and activin A (homo-dimer of INHBA) protein levels were also lower in *FLCN-null *cells compared with *FLCN*-restored cells. Consistent with a growth suppressive function, activin A (but not TGF-β2) completely suppressed anchorage-independent growth of *FLCN-null *UOK257 cells.

**Conclusions:**

Our data demonstrate a role for *FLCN *in the regulation of key molecules in TGF-β signaling and confirm deregulation of their expression in BHD-associated renal tumors. Thus, deregulation of genes involved in TGF-β signaling by *FLCN *inactivation is likely to be an important step for tumorigenesis in BHD syndrome.

## Background

Birt-Hogg-Dubé (BHD) syndrome is a familial disorder that predisposes patients to develop hair follicle hamartomas (84-90% penetrance), lung cysts (85% penetrance) and renal neoplasia (29-34% penetrance) [1-5]. BHD patients are at risk to develop bilateral, multifocal renal tumors with a variety of histologies, mainly chromophobe (34%) and oncocytic hybrid (50%) tumors with features of both chromophobe renal cell carcinoma (RCC) and renal oncocytoma. Clear cell and papillary RCC as well as renal oncocytomas are also found in BHD patients at a low frequency [6]. The BHD syndrome locus was mapped to chromosome 17p11.2 by linkage analysis in BHD families, and germline mutations in a novel gene *FLCN *(alias *BHD*), were identified and characterized [5,7-11]. Most BHD families carry germline mutations predicted to truncate the encoded protein, folliculin (FLCN), including insertion/deletion, nonsense, and splice-site mutations reported in several large BHD cohorts [4,5,11]. Either somatic "second hit" mutations predicted to truncate the protein or loss of heterozygosity at the BHD syndrome locus was identified in 70% of renal tumors from BHD patients [12] supporting a tumor suppressor function for *FLCN*.

Two naturally-occurring animal models have been described that show phenotypes similar to BHD patients. The Nihon rat model develops renal carcinoma with clear cell histology by 6 months of age and harbors a cytosine insertion mutation in exon 3 of rat *Flcn *[13]. A canine model of BHD, which develops renal cystadenocarcinoma and nodular dermatofibrosis (RCND), carries a germline missense mutation (H255R) in canine *Flcn *[14]. Recently, we and others described a conditional *Flcn *knockout mouse model in which *Flcn *inactivation was targeted to mouse kidney using the Cre-lox site-specific recombination system. The affected mice displayed renal hyperplasia, formation of multiple cysts and renal dysfunction, suggesting important roles for *Flcn *in regulation of renal cell proliferation [15,16]. No tumors formed before the animals died at 3 weeks of age due to renal failure, and therefore the mechanism by which *Flcn *inactivation leads to kidney cancer could not be examined in this *in vivo *model. However, recently we and others have reported that mice heterozygous for *Flcn *develop renal cysts and tumors as they age beyond a year [17-19], with demonstrated loss of the wild type copy of *Flcn *(17). These *Flcn *+/- mouse models more closely mimic BHD syndrome in the human, albeit with a long latency.

*FLCN *encodes a 64 kDa protein with no characteristic functional domains, which forms a complex with novel folliculin-interacting proteins 1 and 2 (FNIP1 and FNIP2), and 5'-AMP-activated protein kinase (AMPK), an important energy sensor in cells that negatively regulates mammalian target of rapamycin (mTOR) [20,21]. Phosphorylation of FLCN and FNIP1 was regulated by AMPK and mTOR activities suggesting a functional relationship with the AMPK-mTOR pathway. Interestingly, activation of mTOR downstream signaling molecules was seen in kidney-targeted *BHD *conditional knockout mouse kidneys [15,16]. In addition, the renal tumors from BHD patients showed increased phosphorylation of mTOR [15]. In contrast to these results, it was suggested that yeast homologs of *FLCN *and *TSC1/2 *may have opposing roles in amino acid homeostasis [22]. The cysts and renal tumors derived from the *Flcn *heterozygous mice described by Hartman et al. showed reduced phospho-S6R suggesting diminished mTOR activation [18]. On the other hand Hasumi and coworkers found upregulation of both mTORC1 and mTORC2 pathways in kidney tumors from *Flcn*^*d/+ *^mice [17]. Hudon et al. suggest that up or down regulation of mTOR by inactivation of *Flcn *in a mouse model may be context-dependent [19]. Thus it is possible that mTOR signaling is regulated differently by FLCN depending on cell types or experimental conditions.

A renal cancer cell line (UOK257) established from a BHD patient was recently developed and characterized [23]. UOK257 cells harbor a cytosine insertion in a (poly)C tract, the frequently mutated "hot spot" within exon 11 of *FLCN *(c.1285dupC), and have lost the wild-type copy of *FLCN*. Cytogenetic analysis revealed that the cell line was nearly triploid displaying multiple unbalanced translocations and deletions of chromosomes. The MYC copy number was heterogeneous in UOK257 cells ranging from 3 to 5 copies. These cells formed tumors in immunodeficient mice (SCID/BEIG) exhibiting predominantly atypical clear epithelial cell type histology, as well as a variety of other histologic types including tubular papillary, and foci reminiscent of chromophobe RCC, all of which resemble the histologies within the tumor from which the cell line was derived [23].

In the current study, in order to investigate the tumor suppressor function of *FLCN *we have introduced wild-type *FLCN *into UOK257 cells and compared their growth *in vitro *and *in vivo*. We found that wild-type *FLCN *suppressed tumor cell growth *in vivo*, confirming the tumor suppressor function of *FLCN*. In addition, we employed gene expression microarray analysis to identify novel downstream target genes of *FLCN*. Among the differentially expressed genes, we identified several critical genes involved in TGF-β signaling including *TGFB2*, *INHBA*, *THBS1*, *GREM1 *and *SMAD3*. Since deregulation of TGF-β signaling is important in tumorigenesis and tumor progression, we characterized the expression of these genes in *FLCN*-*null *and *FLCN*-expressing cultured cells as well as in renal tumors surgically removed from BHD patients. In addition, we examined the growth suppressive effect of activin A in the *FLCN-null *cell line and investigated receptor mediated TGF-β signaling in *FLCN-null *and *FLCN*-restored cell lines.

## Results

### Wild-type *FLCN *expression was restored in UOK257 cells by lentiviral vectors

**Figure 1:**
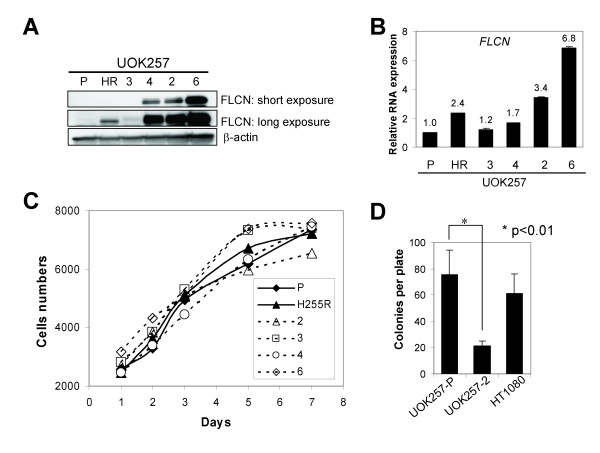
**Characterization of the UOK257 cell lines**. (A) FLCN protein expression in the UOK257 cell lines restored with wildtype or mutant *FLCN*. P, parental; HR, FLCN (H255R) mutant; 2, 3, 4 and 6, wildtype *FLCN*. (B) *FLCN *mRNA level was measured by quantitative RT-PCR. *Columns*, mean; *bars*, +SD (n = 3). (C) Cell growth of the UOK257 cell lines *in vitro*. Points, mean (n = 4). (D) Colony formation assay of the UOK257 cell lines and control HT1080 cell line in soft-agar culture. Columns, mean; bars, +SD (n = 3).

To evaluate the tumor suppressor function of *FLCN*, wild-type or mutant (H255R) *FLCN *cDNA was introduced into the *FLCN*-null UOK257 cells using lentiviral vectors. Four clones expressing wild-type *FLCN *(UOK257-2, -3, -4, and -6) and one clone expressing mutant *FLCN-H255R *(UOK257-H255R; *Flcn *missense mutation responsible for canine RCND) were isolated and compared to the parental UOK257 cells (UOK257-P). FLCN protein expression was measured by Western blot analysis using a mouse monoclonal anti-FLCN antibody. Relatively high levels of FLCN protein were detected in the UOK257-2, -4 and -6 cells but very low levels of FLCN protein were detected in the UOK257-3 and UOK257-H255R cells (Fig. 1A). *FLCN *mRNA expression from both the transgene and endogenous *FLCN *was measured by quantitative RT-PCR. The total *FLCN *mRNA expression was increased by the expression of the wild-type *FLCN *or mutant *FLCN-H255R *transgene to varying degrees in the cell lines (P = 3 < 4 < H255R <2 <6) (Fig. 1B).

### Anchorage independent but not dependent growth of UOK257 cells was inversely correlated with wild-type *FLCN *expression

We examined whether introduction of wild-type or mutant *FLCN *could affect anchorage dependent and independent growth of UOK257 cells. Anchorage dependent growth of UOK257 cells on culture dishes was not affected by the expression of wild-type or mutant *FLCN *(Fig. 1C). However, anchorage independent growth measured as colony numbers on soft agar was low in the wild-type *FLCN *cell line UOK257-2, which expressed high levels of *FLCN*, compared to the parental UOK257 cell line (UOK257-P) (Fig. 1D). One of the characteristics of the UOK257 cells was a slow growth rate (doubling time, 52+/- 9 hrs) on culture dishes. These cells also grew very slowly in soft agar taking 3-4 weeks to reach a countable colony size. By comparison, HT-1080 cells derived from a fibrosarcoma grew faster in soft agar and often generated larger colonies (data not shown).

### Tumor growth was suppressed by wild-type *FLCN *but not by mutant *FLCN-H255R *expression

**Figure 2:**
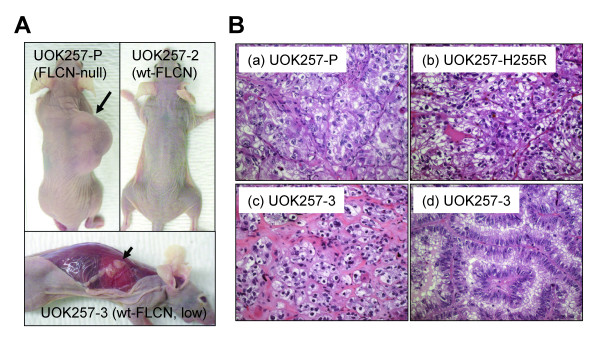
**Suppression of tumorigenesis by re-introduction of wild-type *FLCN *not mutant *FLCN *in the *FLCN*-null cell line, UOK257**. (A) Tumorigenesis of UOK257 cells in nude mice was suppressed by wild-type *FLCN*. Arrow indicates a tumor from UOK257-P cells. Arrowhead indicates a flat patch of tumor cells from UOK257-3 cells. (B) Histologies of the tumors from different clones of UOK257 cells. (a) high, (b) medium, and (c) low grade clear cell histologies; (d) papillary RCC.

To examine whether the tumorigenic potential of UOK257 cells was affected by wild-type or mutant *FLCN*, mutant *FLCN *(UOK257-P and -H255R) and wild-type *FLCN*-expressing cells (UOK257-2, -3, -4 and -6) were injected subcutaneously with matrigel into athymic nude mice and tumor growth was measured for up to one year. Most of the mice injected with UOK257-P and UOK257-H255R cells developed solid tumors, although some animals only developed flat patches of tumor cells (See additional file 1: Table S1 and Fig. 2A). All of these tumors were high grade and exhibited clear cell histology (See additional file 1: Table S1 and Fig. 2B). On the other hand, the mice injected with UOK257 cells expressing a high level of *FLCN *(UOK257-2, -4 and -6) did not develop tumors (See additional file 1: Table S1 and Fig. 2A). Instead, flat masses of cells only rarely containing tumor cells were observed in 6 of 35 (17%) of the animals. The mice injected with UOK257-3 cells expressing a very low level of *FLCN *developed solid tumors with low incidence (2/10) and smaller size (See additional file 1: Table S1). In some animals, UOK257-3 cells grew as flat patches (5/10; Fig. 2A) and exhibited mostly clear cell histology with varying grades (low to high; Fig. 2B).

We investigated whether wild-type or mutant *FLCN *transgenes, or the endogenous mutant *FLCN *genes were lost during tumor progression. Genomic DNA was isolated from the tumors or tumor cell patches and PCR was performed using a primer pair specific to exon 10 and exon 11 that amplifies 664 bp of the endogenous *FLCN *gene or 99 bp of the *FLCN *transgene. All of the tumors from the cell lines retained the endogenous mutant *FLCN *gene (c.1285dupC) and all of the tumors from UOK257-3 and UOK257-H255R retained their respective transgenes (See additional file 1: Fig. S1).

### Gene expression microarray analysis identified genes regulated by *FLCN *and the pathways in which they interact

**Figure 3:**
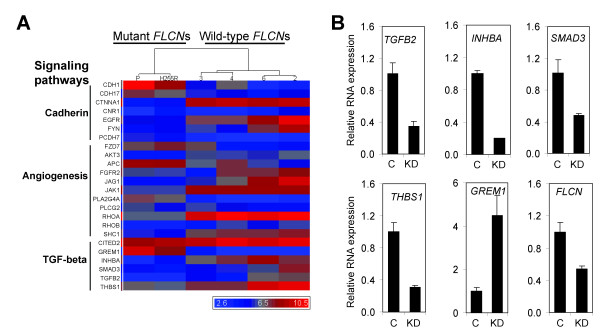
**Genes differentially expressed in mutant and wild-type *FLCN *cell lines**. (A) Heat-map representation of the genes differentially expressed in mutant and wild-type *FLCN *cell lines, and hierarchical clustering of the cell lines. Gene symbols are listed on the left side of each row. Three prominent groups of genes involved in signaling pathways were indicated on the left side of the gene symbols. (B) Deregulation of the key molecules in TGF-β signaling by *FLCN *knockdown in the UOK257-2 cells expressing wildtype FLCN. C, control cell line infected with empty retrovirus; KD, *FLCN*-knockdown cell line infected with a retrovirus expressing short hairpin RNA targeting *FLCN*.

To identify the genes regulated by *FLCN *expression, we performed gene expression microarray analysis using RNAs isolated from the UOK257 cell lines expressing either no, mutant or wild-type *FLCN*. We identified a total of 439 genes, which were up or down-regulated more than 2-fold in the mutant and *FLCN*-*null *cell lines (UOK257-P and UOK257-H255R) compared to the wild-type *FLCN *cell lines (UOK257-2, -3, -4 and -6) (See additional file 2: Table S2). To explore the biological processes and pathways regulated by *FLCN*, the genes were subclassified with the help of "Panther Classification System" http://www.pantherdb.org and three prominent pathways were identified, namely cadherin signaling, TGF-β signaling, and angiogenesis (Fig. 3A). Although all three of these pathways are important in tumorigenesis, we focused on the genes involved in TGF-β signaling (See additional file 1: Fig. S2). We found that *TGF-β2 *(*TGFB2*), *Inhibin β A *(*INHBA*, a subunit of activin A), *SMAD3 *(*SMAD3*) and *thrombospondin-1 *(*THBS1*) were down-regulated, and *Gremlin *(*GREM1*) was upregulated in *FLCN*-*null *and mutant *FLCN-H255R *UOK257 cells compared with *FLCN*-restored UOK257 cells. We confirmed the *GREM1, TGFB2, INHBA, SMAD3 *and *THBS1 *microarray results by quantitative RT-PCR (See additional file 1: Fig. S3).

### Knockdown of *FLCN *deregulates *TGFB2, INHBA, GREM1, THBS1 *and *SMAD3 *expression in *FLCN*-restored UOK257 cells

We next examined whether the expression levels of *TGFB2*, *INHBA*, *THBS1*, *GREM1 *and *SMAD3 *could be deregulated by knockdown of *FLCN *in *FLCN*-restored UOK257 cells. A *FLCN-*knockdown cell line was generated by introducing a retrovirus that expressed shRNA against *FLCN *in *FLCN*-restored cells (UOK257-2). In addition to reduced expression of *FLCN*, the expression of *TGFB2*, *INHBA*, *THBS1 *and *SMAD3 *was decreased and the expression of *GREM1 *was increased in the *FLCN*-knockdown cell line (Fig. 3B).

### *GREM1*, *TGFB2*, *INHBA*, *THBS1 *and *SMAD3 *expression levels were down-regulated in BHD-associated renal tumors

**Figure 4:**
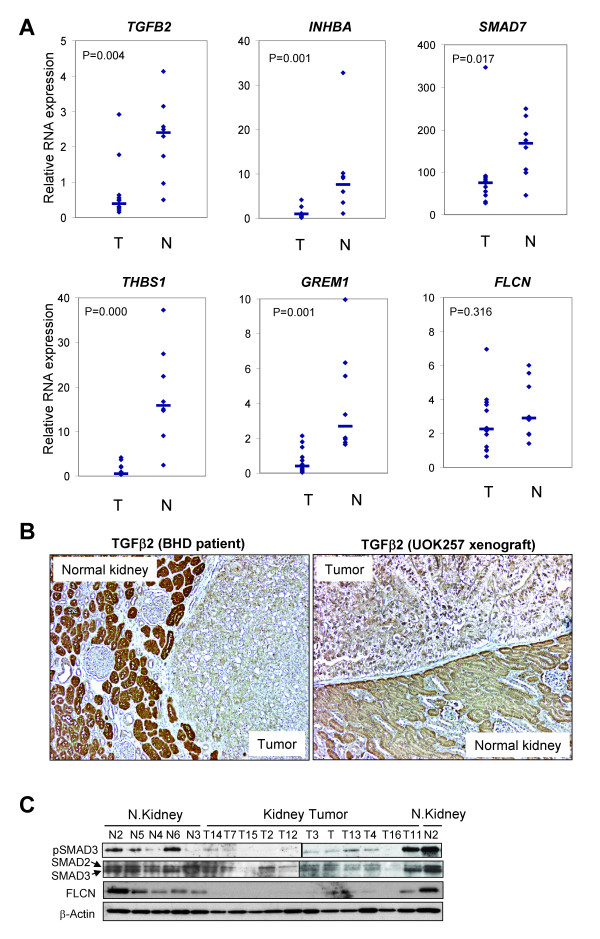
**Genes involved in TGF-β signaling and the encoded proteins dysregulated in renal tumors from BHD patients**. (A) Quantitative RT-PCR of *TGFB2*, *INHBA*, *SMAD3*, *THBS1*, *GREM1 *and *FLCN *expression levels in human BHD tumors (T, n = 12) and normal kidneys (N, n = 8). Gene expressions were normalized against cyclophilin gene expression and relative expressions were calculated against gene expression in UOK257 cells. Median values of expression levels were indicated with short lines. Points, mean expression of each sample. P value, Mann-Whittney U-test. (B) Reduced expression of TGF-β2 in the BHD tumors (left panel) and the UOK257 xenograft tumors (right panel) compared to normal renal tubules as shown by immunohistochemical staining. Five BHD tumors were examined and a representative immunostained image is shown. (C) Positive correlation of pSMAD3 and SMAD3 with FLCN expression in human BHD kidney tumors and normal kidney tissue. pSMAD3, SMAD2, SMAD3 and FLCN protein levels were measured by western blot analysis in normal kidney tissue (n = 5) and BHD tumors (n = 11).

In order to determine whether the genes that were regulated by *FLCN *in *in vitro *cell culture were differentially expressed in renal tumors from BHD patients compared to normal kidney parenchyma, we performed quantitative RT-PCR using RNA isolated from these tissues. *GREM1, TGFB2*, *INHBA*, *THBS1 *and *SMAD3 *RNA expression levels were significantly lower in the BHD renal tumors compared to normal kidney tissue (Fig. 4A). However, *FLCN *RNA levels were not statistically different (P = 0.316). In support of the RT-PCR data, immunohistochemical staining of TGF-β2 showed strong TGF-β2 expression in the normal renal tubules but reduced expression in the tumors from BHD patients (Fig. 4B, left panel). In addition, the UOK257 xenograft tumors expressed lower levels of TGFB2 compared to normal mouse kidney (Fig. 4B, right panel).

We measured protein expression of SMAD2, SMAD3, phospho-SMAD3 (pSMAD3) and FLCN in renal tumors from BHD patients (n = 11) and normal human kidney tissue (n = 5). pSMAD3 levels were high in 3 out of 5 normal kidneys but only 1 (T11) of 11 tumors (Fig. 4C). In addition, SMAD3 levels and SMAD3/SMAD2 ratios were higher in normal kidneys compared to the tumors. On the other hand FLCN protein levels were lower or undetectable in all tumors except T11, in which a moderate level of FLCN expression was detected along with high levels of pSMAD3 and SMAD3 (Fig. 4C). Therefore it is likely that the T11 tumor was contaminated with normal kidney tissue.

**Figure 5:**
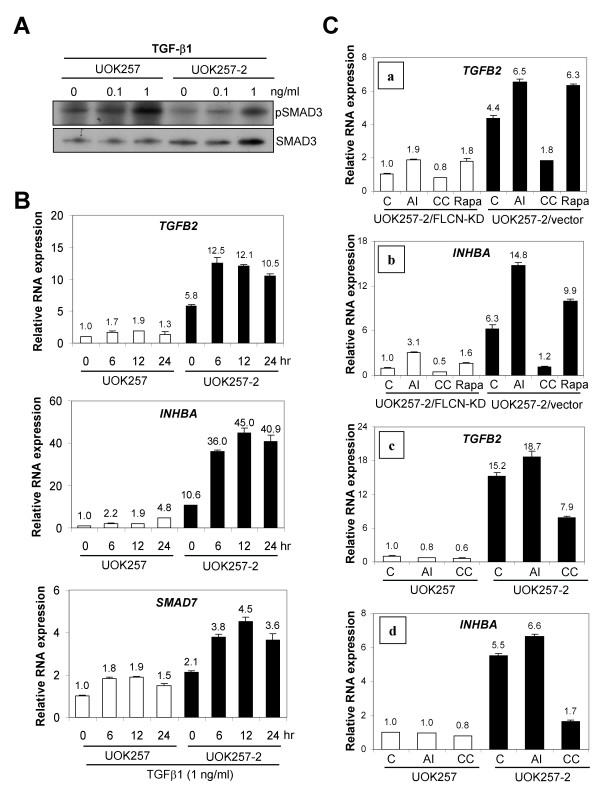
**TGF-β1 induced SMAD3 phosphorylation and effects of AICAR, Compound C and rapamycin on TGF- β1 induction of *TGFB2 *and *INHBA *in *FLCN-null *and *FLCN*-expressing cells**. (A) TGF-β1 induced SMAD3 phosphorylation in UOK257 and UOK257-2 cells. Cells were treated with 0, 0.1 and 1 ng/ml of TGF-β1 for 1 hr. (B) TGF-β1 induced *TGFB2*, *INHBA *and *SMAD7 *mRNA expression in UOK257 and UOK257-2 cells. Cell were treated with 1 ng/ml of TGF-β1 for 0, 6, 12 and 24 hr after serum starvation for 12 hr. (C) (a and b) Cells were serum-starved for 24 hr and treated with 0.5 mM AICAR (AI), 20 μM Compound C (CC) or 1 nM rapamycin (Rapa) for 12 hr. UOK257-2/vector, UOK257-2 cells infected with empty retrovirus; UOK257-2/FLCN-KD, UOK257-2 cells infected with retrovirus expressing shRNA targeting *FLCN*. (c and d) Cells were serum-starved for 15 hr and treated with 0.5 mM AICAR or 20 μM Compound C (CC) for 6 hr.

In order to investigate whether receptor mediated TGF-β signaling was disrupted by the loss of FLCN expression, TGF-β induced SMAD3 phosphorylation was examined in UOK257 cells and compared to UOK257-2 cells. TGF-β induced SMAD3 phosphorylation was not affected by FLCN inactivation (Fig. 5A). In addition, BMP4 induced SMAD1/5/8 phosphorylation was not disrupted by loss of FLCN expression (data not shown). We then examined whether TGF-β induced gene expression was dysregulated in *FLCN-null *UOK257 cells. *SMAD7*, an inhibitory SMAD, is known to be induced by TGF-β. *SMAD7 *expression was induced in both UOK257 and UOK257-2 cell lines (Fig. 5B). However the basal and the maximal induced levels of *SMAD7 *were two fold greater in UOK257-2 cells than in UOK257 cells. Similar to *SMAD7*, *TGFB2 *and *INHBA *expressions were induced by TGF-β in both cell lines but their basal and maximal levels of expression were substantially higher (5.8- to 23-fold) in *FLCN*-restored UOK257-2 cells compared with *FLCN-null *UOK257 cells (Fig. 5B).

Since FLCN and FNIP1/2 can complex with AMPK, and phosphorylation of these proteins is affected by AMPK and mTOR signaling, we wanted to know whether AMPK and mTOR signaling affected the expression of *TGFB2 *and *INHBA *in a *FLCN *-dependent manner. Interestingly, both *TGFB2 *and *INHBA *were induced by the AMPK activator AICAR but reduced by the AMPK inhibitor Compound C in UOK257-2 cells expressing *FLCN *as well as in cells in which *FLCN *expression was knocked down by a retrovirus expressing *FLCN *shRNA [UOK257-2/FLCN-KD; Fig. 5C(a) and Fig. 5C(b)]. Rapamycin, an inhibitor of mTOR signaling, also induced *TGFB2 *and *INHBA *expression in both *FLCN*-expressing UOK257-2 and UOK257-2/FLCN-KD cells [Fig. 5C(a) and Fig. 5C(b)]. However, the basal and maximal levels of induction of *TGFB2 *and *INHBA *by AICAR and rapamycin were higher in *FLCN*-expressing UOK257-2 cells compared to UOK257-2/FLCN-KD cells [Fig. 5C(a) and Fig. 5C(b)]. Similar results were obtained from an experiment using UOK257 *FLCN-null *and UOK257-2 *FLCN*-expressing cell lines. Interestingly, AICAR induced *TGFB2 *and *INHBA *expression in UOK257-2 cells but not in UOK257 cells [Fig. 5C(c) and Fig. 5C(d)].

### Activin A but not TGF-β2 suppressed anchorage-independent growth of UOK257 cells

**Figure 6:**
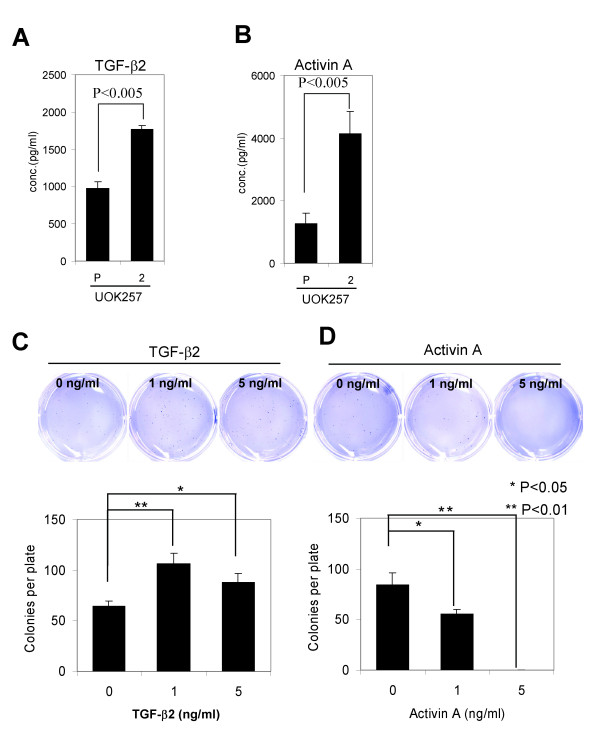
**Higher levels of secreted TGF-β2 and activin A proteins were detected in *FLCN*-restored UOK257-2 cells compared to the parental UOK257 cell line**. (A) TGF-β2 and (B) activin A levels in cultured media were measured by ELISA (R&D systems). (C) TGF-β2 induced anchorage independent growth of UOK257 cells. However, (D) activin A suppressed anchorage independent growth of UOK257 cells. UOK257 cells (2,500 cells) were plated in soft-agar and cultured for 4 weeks in the presence of TGF-β2 or Activin A and stained with crystal violet. N = 4 for each treatment.

Initially, in order to confirm that protein expression levels were consistent with the mRNA expression levels, we measured secreted TGF-β2 and activin A levels in the media of UOK257 and UOK257-2 cells by ELISA. In accordance with their mRNA expression, TGF-β2 and activin A protein expression levels were lower in UOK257 cells compared to UOK257-2 cells (Fig. 6A and 6B). Since both TGF-β2 and activin A have been reported to suppress cell growth, we examined their effect on growth of UOK257 cells. To evaluate the growth suppressive effects of TGF-β2 and activin A, UOK257 cells were treated with TGF-β2 or activin A and cultured for 4 weeks in soft-agar. Unexpectedly, TGF-β2 appeared to increase colony formation of UOK257 cells at both 1 ng/ml and 5 ng/ml (Fig. 6C). However, activin A reduced colony formation at 1 ng/ml and completely suppressed colony formation at 5 ng/ml (Fig. 6D).

## Discussion

UOK257 is the only renal cancer cell line available to date that has been established from a BHD patient's tumor tissue. This cell line is particularly valuable for study of the biological role of *FLCN *inactivation in tumorigenesis because it harbors a *FLCN *mutation predicted to produce only truncated mutant protein and induces the growth of tumors *in vivo *with histology resembling the BHD-associated renal tumor from which it was derived [23]. In this study, we have established and characterized UOK257 cell lines in which wild-type or mutant *FLCN *was stably expressed. Although anchorage dependent cell growth *in vitro *was not affected by wild-type *FLCN *expression, cell growth *in vivo *and anchorage-independent growth in soft agar were severely diminished by the expression of wild-type *FLCN*. We have searched for downstream target genes regulated by *FLCN *through gene expression microarray analysis and identified a number of genes that were differentially expressed in wild-type *FLCN *(UOK257-2, -4, and -6) compared with mutant *FLCN *and *FLCN*-null (UOK257-H255R and -P) cells. We found three prominent groups of genes involved in cadherin signaling, TGF-β signaling, and angiogenesis. Notably, several key genes involved in TGF-β signaling, such as *TGFB2*, *INHBA*, *THBS1 *and *SMAD3*, were down-regulated in *FLCN*-null and mutant *FLCN *cells as well as in the BHD-associated renal tumors. Consistently, *GREM1*, the antagonist of BMP that signals through SMADs was highly up-regulated in mutant *FLCN *and *FLCN*-null UOK257 cells although its expression was low in BHD-associated renal tumors.

We observed that the expression level of FLCN is important for tumor suppression, since the UOK257 cell lines (UOK257-4, -2 and -6) expressing high levels of FLCN did not develop tumors whereas the UOK257-3 cell line expressing a very low level of FLCN, did develop tumors with a low incidence (2 out of 10). It is likely that the FLCN expression level in UOK257-3 cells is marginal for tumor suppression, allowing tumor growth in some animals but suppressing tumor growth in others. In support of this idea, the expression levels of the downstream target genes in UOK257-3 cells were either similar to *FLCN-null *and *FLCN *mutant cells (UOK257-P and UOK257-H255R), or midway between the *FLCN-null*-*FLCN *mutant group and the *FLCN*-restored group, which expressed high levels of FLCN (UOK257-2 and UOK257-6) (See additional file 1: Fig. S3).

UOK257-H255R cells expressed a low level of FLCN protein resulting in loss of tumor suppressor function and deregulation of TGF-β signaling, even though they expressed slightly more *FLCN *mRNA than UOK257-4 cells (Fig.1A and 1B). These data suggest that FLCN-H255R missense mutant protein found in the canine model of BHD syndrome is less stable than wild-type FLCN. Thus decreased stability of mutant FLCN is likely to contribute to the loss of FLCN tumor suppressor function.

It has been suggested that *Drosophila BHD *(*dBHD*) regulates germline stem cell (GSC) maintenance downstream or in parallel with Jak/Stat and dpp (BMP ortholog in *Drosophila*) signaling [24]. *dBHD *knockdown by siRNA suppressed overproliferation of GSC induced by hyperactivation of Jak/Stat or dpp signaling. Interestingly, *Jak1*, encoding a kinase that transmits signals by phosphorylating Stats in cells, was identified by microarray analysis as a downregulated gene in the mutant *FLCN *and *FLCN*-null cells (Fig. 3A). We also identified several key genes in TGF-β/BMP signaling such as *TGFB2*, *INHBA*, *THBS1 *and *SMAD3 *(a regulatory SMAD) that were down-regulated in the mutant *FLCN *and *FLCN-null *cells. On the other hand, *GREM1*, which encodes a protein that binds and inactivates BMP activity, was upregulated in the mutant and *FLCN-null *cells. Thus the genetic interactions between *dBHD*, and Jak/Stat and dpp (BMP) signaling may be partially explained by *FLCN *deregulation of genes involved in these pathways.

The human TGF-β superfamily consists of 42 members including TGF-βs, activins, bone morphogenic proteins (BMPs), and growth and differentiating factors (GDFs) [25,26]. TGF-βs are multi-functional cytokines that modulate cell proliferation, apoptosis, differentiation, adhesion and migration. TGF-β shows a biphasic effect on tumor cell growth [27]. It inhibits tumor cell growth in the early phase of tumorigenesis but promotes cell growth when cells escape the anti-proliferative effect of TGF-β in the late phase of tumorigenesis. Interestingly, TGF-β2 induced anchorage independent growth of UOK257 cells (Fig. 6C), suggesting that UOK257 cells are refractory to the growth suppressive effect of TGF-β. The possibility exists that reduced expression of TGF-β2 in *FLCN*-null cells contributed to cell growth in the early phase of tumorigenesis.

Disruption of TGF-β signaling has been reported in many cancers. TGF-β type II receptor is often mutated in gastro-intestinal cancers [28-30]. Mutations in SMAD2 or SMAD4 occur frequently in pancreatic and colorectal carcinomas [31-33]. Although mutations in SMAD3 have not been reported, 3 out of 8 (37.5%) gastric tumors in one report showed low to undetectable levels of SMAD3 expression and restoration of SMAD3 suppressed tumorigenicity of gastric cancer cells [34]. Low levels of SMAD3 expression in the BHD tumors may contribute to the ability of these renal tumor cells to escape the growth suppressive effect of TGF-β.

Activins are homo- or heterodimeric proteins consisting of two β subunits (βA and βB), while inhibins are heterodimers of α and β subunits (inhibin-A [αβA] and inhibin-B [αβB]) [35]. INHBA is one of the β subunits (βA) that comprise activin A (βAβA), activin AB (βAβB) and inhibin A (αβA). Activin A regulates kidney organogenesis, tubular regeneration and renal fibrosis [reviewed in [36]]. Activins also induce apoptosis, and inhibit cell proliferation and tumor growth in numerous types of cells. In contrast to TGF-β2, activin A inhibited growth of UOK257 cells in soft-agar (Fig. 6D), suggesting that activin signaling is intact in UOK257 cells. Thus reduced expression of *INHBA*, β subunit of activin A, in UOK257 cells and BHD tumors, may be permissive for tumor cell growth. It would be interesting to examine whether activin A treatment can suppress BHD tumor growth *in vivo*.

Thrombospondin-1 (THBS1) is one of the five members of a family of thrombospondins that mediate the interaction of normal and cancer cells with the extracellular matrix and surrounding tissue. THBS1 suppresses tumor growth by activating TGF-β and by inhibiting angiogenesis. THBS1 exerts direct effects on endothelial cell migration and survival through interaction with CD36. It also reduces availability of VEGF by inhibiting MMP9, therefore releasing VEGF from the extracellular matrix. There are several reports suggesting that reduced expression of THBS1 or hypermethylation of *THBS1 *is associated with poor prognosis of cancer patients and higher tumor grade [37-40]. Accordingly THBS1 regulation may be an important part of the tumor suppressor function of *FLCN*.

We examined whether TGF-β signaling is dysregulated by the inactivation of the *FLCN *gene. TGF-β or BMP4 induced SMAD3 or SMAD1/5/8 phosphorylation was not affected by FLCN inactivation suggesting receptor mediated SMAD phosphorylation is not altered by FLCN. However, several genes whose expressions are regulated by TGF-β were dysregulated by the inactivation of FLCN. The basal and maximal induced levels of the downstream target genes (*TGFB2*, *INHBA *and *SMAD7*) regulated by TGF-β were reduced in cells with FLCN inactivation. These data suggest that FLCN may regulate TGF-β signaling through a non-SMAD mediated mechanism. As a result of such regulation, the level of TGF-β ligands, such as TGF-β2 and activin A, could be highly induced in cells expressing *FLCN *by a positive feedback control.

A possible function of FLCN in energy sensing and metabolism has been suggested by its interaction with AMPK through FNIP1/2 and by the observation that FLCN phosphorylation is affected by mTOR signaling (20-21). Here we demonstrated that an AMPK activator, AICAR, and an AMPK inhibitor, Compound C, as well as an mTOR inhibitor, rapamycin, affected the expression of the same key molecules involved in TGF-β signaling, which appear to be regulated by FLCN. Thus FLCN could be a key molecule connecting energy-sensing signals to growth suppressive TGF-β signaling.

## Conclusions

Here for the first time we have confirmed the tumor suppressor function of *FLCN in vivo *and identified new potential FLCN downstream targets in the TGF-β signaling pathway. This study will provide a foundation for understanding the pathogenesis of BHD syndrome at the molecular level and be useful for finding therapeutic targets for treating BHD-associated kidney cancer and potentially, sporadic chromophobe RCC. We are currently analyzing the mechanism by which FLCN regulates these target genes and the functional importance of deregulation of these FLCN target genes in tumorigenesis.

## Methods

### Establishment of cell lines, cell culture, and cell growth

Wild-type or mutant (H255R) *FLCN *cDNA was transduced into UOK257 cells using the ViraPower Lentiviral expression system (Invitrogen) following the manufacturer's protocols. Stable clones were selected using Blasticidin S (1.5 μg/ml). Cells were maintained in DMEM medium supplemented with 10% fetal bovine serum (FBS) and penicillin/streptomycin. To evaluate growth rate in culture, cells (2 × 10^3^) were plated in each well of five 96 well plates, cultured, and cell numbers were measured at day 1, 2, 3, 5 and 7 using the CyQuant Cell Proliferation Assay Kit (Molecular Probes). Adenoviral vectors (pAd/CMV/V5-DEST) expressing wild-type and mutant (c.1285dupC) *FLCN *were generated using the ViraPower Adenoviral Gateway system (Invitrogen) following the manufacturer's protocol. A retroviral shRNA vector targeting *FLCN *was generated by inserting double stranded oligonucleotides (forward sequence, 5'-GATCCCCGGTGTTTGAGGCAGAGCAGTTCAAGAGACTGCTCTGCCTCAAACACCTTTTTA-3' and reverse sequence, 5'-GCTTAAAAAGGTGTTTGAGGCAGAGCAGTCTCTTGAACTGCTCTGCCTCAAACACCGGG-3') into HindIII and BglII sites of pSuper-Retro vector (Oligoengine) following the manufacturer's instruction. UOK257-2 cells were infected with the *FLCN *shRNA vectors and selected against puromycin (7.5 ug/ml).

### Colony formation assay

UOK257 cells (5 × 10^3^) were suspended in 1.5 ml of 0.3% agar in DMEM containing 10% FBS and were overlayed on 1.5 ml of pre-solidified 0.5% agar in the same medium. Cells were cultured in a CO_2 _incubator for 3-4 weeks. Colonies were stained for 1 hour with 0.02% crystal violet solution dissolved in 10% neutral formalin. Colony number was counted under a dissection microscope after washing with PBS three times.

### Tumor growth in nude mice

Cells (1 × 10^6^) suspended with basement membrane matrix (BD Biosciences) were injected subcutaneously into the flanks of athymic nude mice. Tumor growth was measured once a week and mouse health was monitored daily. Mice bearing tumors larger than 2 cm, or showing severe health problems, were sacrificed and examined. Otherwise tumor growth was monitored for up to one year after injection. Tumors were fixed in 10% buffered formalin solution for histological examination and flash frozen in liquid nitrogen for protein and RNA extraction. Animal care procedures followed NCI-Frederick Animal Care and Use Committee guidelines.

### Immunoblotting

Cells were harvested and lysed in RIPA buffer (50 mM Tris-Cl, pH 8.0 with 150 mM NaCl, 1.0% NP-40 and 0.5% sodium deoxycholate) or 1× SDS sample buffer (Biorad). Cell lysates were resolved by 4-20% SDS PAGE and blotted onto PVDF membrane. The following antibodies were used in this study: anti-FLCN mouse monoclonal [20], anti-β-actin (Sigma), anti-SMAD2/3 (Santa Cruz, sc-6032), and anti-pSMAD2/3 (Santa Cruz, sc-11769) antibodies. Immunoblots were processed by the ECL Detection System (Pierce) according to the manufacturer's protocols.

### Immunohistochemistry

Paraffin tissue sections were deparaffinized, rehydrated in graded alcohol and boiled in Tris-EDTA buffer pH 8.0 for 20 min at 90°C for antigen retrieval. After blocking, sections were probed with primary antibodies overnight and then incubated with HRP-polymer conjugated secondary antibodies. Diaminobenzidine hydrochloride (DAB) was used as a substrate for peroxidase. Sections were then briefly counterstained with hematoxylin and permanently mounted for observation.

### ELISA

Cells (2 × 10^5^) were cultured on 6 well plates for 3 days and culture media was collected for assay. TGF-β2, and activin A levels in the media were quantified by Human TGF-β2 DuoSet (R&D systems) and activin A DuoSet (R&D systems), respectively, following the manufacturer's instruction.

### RNA isolation, microarray analysis and pathway analysis

Total RNAs were isolated from the UOK257 cell lines using Trizol reagent (Invitrogen) and further purified using RNeasy mini kit (QIAGEN) according to the manufacturer's protocols. Probes, which were generated using these RNAs, were hybridized to the Human Genome U133 Plus 2.0 arrays (Affymetrix) and processed according to recommended protocols. The CEL files were processed using the Partek Genomic Suite 6.2 (Partek Inc.). Data were transformed using a log normalization process and the differentially expressed genes were identified by Student's t-test and Mann-Whitney U-test. The genes that were differentially expressed in mutant *FLCN *cell lines (UOK257-P and -H255R) and wild-type *FLCN *cell lines (UOK257-2, -4 and -6) were used for further analysis.

### Quantitative real-time reverse transcription-PCR (RT-PCR)

To confirm the microarray results, quantitative real-time reverse transcription PCR (RT-PCR) was performed. RNAs were digested with DNase I for 30 min at 37°C followed by heat denaturation at 70°C for 20 min to remove genomic DNA contamination. Total RNAs (2.5 μg) were primed with 100 ng random primers and reverse-transcribed by Superscript II reverse transcriptase (Invitrogen) at 42°C for 1 hr. The identical reactions were performed without reverse transcriptase to generate negative controls. PCR primers were generated using Primer 3 software [41] or Primer Express 3.0 (Applied Biosystems). Quantitative RT-PCR was performed with Power SYBR-Green or Taqman Gene Expression Master Mix (Applied Biosystems) using a 7300 Real-Time PCR system (Applied Biosystems) following the manufacturer's protocols. All reactions were run in triplicate using *β-actin*, *GAPDH *or *cyclophilin A *genes as internal controls. The relative level of a particular gene expression was evaluated according to the function of 2^-ddCt^, where ddCt is dCt_(treatment) _- dCt_(control)_, dCt is Ct_(target gene) _- Ct_(GAPDH or actin) _and Ct is the cycle at which the threshold is crossed. The gene-specific primer pairs for the PCR reactions are as follows: *FLCN *forward 5'-TTCACGCCATTCCTACACCAGA-3' and reverse 5'-GCCCACAGGTTGTCATCACTTG-3', *GREM1 *forward 5'-GCAAAACCCAGCCGCTTAA-3' and reverse 5'-TGATGGTGCGACTGTTGCA-3', *TGFB2 *forward 5'-CGAGAGGAGCGACGAAGAGT-3' and reverse 5'-AGGGCGGCATGTCTATTTTG-3', *THBS1 *forward 5'-CCAGATCAGGCAGACACAGA-3' and reverse 5'-AGTTGTCCCGTTCATTGAGG-3', *INHBA *forward 5'-TGGAGTGTGATGGCAAGGTCA-3' and reverse 5'-GCATGATAGCCAGAGGGAGCA-3', *SMAD3 *forward 5'-GACGAGGTCTGCGTGAATCC-3' and reverse 5'-GTGGCGTGGCACCAACA-3', and *GAPDH *forward 5'-TTCCACCCATGGCAAATTCC-3' and reverse 5'-CGCCCCACTTGATTTTGGAG-3'. *SMAD7 *forward 5'-CCAACTGCAGACTGTCCAGA-3' and reverse 5'-CAGGCTCCAGAAGAAGTTGG-3'. PCR product quality was monitored using post-PCR dissociation curve analysis.

## Competing interests

The authors declare that they have no competing interests.

## Authors' contributions

SBH designed the experiments. SBH, VAV, HBO, JS, DTN and MB performed the experimental work. MB generated critical cell lines for the work. MJM performed histopathologic analysis. SBH wrote the manuscript. WML and LSS contributed to the design of the experiments, review of the data, scientific discussions and manuscript editing. All authors read and approved the final manuscript.

## Supplementary Material

Additional file 1 **table S1, and figures S1, S2, S3**. Table S1. Frequency and characteristics of tumors that developed from different UOK257 cell lines in athymic nude mice. Figure S1. PCR amplification of endogenous *FLCN *(endo) and *FLCN *transgene (tg) from the xenograft tumors. Figure S2. Deregulation of the key molecules in TGF-β signaling by *FLCN *expression. Figure S3. Quantitative RT-PCR for *TGFB2*, *INHBA*, *THBS1*, *GREM1 *and *SMAD3 *in the UOK257 cell lines expressing either mutant or wild-type *FLCN*. Click here for file

Additional file 2 **Table S2**. Table S2. The genes differentially expressed in the wild-type *FLCN *and mutant *FLCN *cell lines. Click here for file

## References

[B1] Birt AR Hogg GR Dube WJ Hereditary multiple fibrofolliculomas with trichodiscomas and acrochordons Arch Dermatol 1977 113 1674 1677 10.1001/archderm.113.12.1674 596896

[B2] Toro JR Glenn G Duray P Darling T Weirich G Zbar B Linehan M Turner ML Birt-Hogg-Dube syndrome: a novel marker of kidney neoplasia Arch Dermatol 1999 135 1195 1202 10.1001/archderm.135.10.1195 10522666

[B3] Zbar B Alvord WG Glenn G Turner M Pavlovich CP Schmidt L Walther M Choyke P Weirich G Hewitt SM Duray P Gabril F Greenberg C Merino MJ Toro J Linehan WM Risk of renal and colonic neoplasms and spontaneous pneumothorax in the Birt-Hogg-Dube syndrome Cancer Epidemiol Biomarkers Prev 2002 11 393 400 11927500

[B4] Schmidt LS Nickerson ML Warren MB Glenn GM Toro JR Merino MJ Turner ML Choyke PL Sharma N Peterson J Morrison P Maher ER Walther MM Zbar B Linehan WM Germline *BHD*-mutation spectrum and phenotype analysis of a large cohort of families with Birt-Hogg-Dube syndrome Am J Hum Genet 2005 76 1023 1033 10.1086/430842 15852235 PMC1196440

[B5] Toro JR Wei MH Glenn G Weinreich M Toure O Vocke C Turner M Choyke P Merino MJ Pinto PA Steinberg SM Schmidt LS Linehan WM *BHD *mutations, clinical and molecular genetic investigations of Birt-Hogg-Dube Syndrome: A new series of 50 families and a review of published reports J Med Genet 2008 45 321 331 10.1136/jmg.2007.054304 18234728 PMC2564862

[B6] Pavlovich CP Walther MM Eyler RA Hewitt SM Zbar B Linehan WM Merino MJ Renal tumors in the Birt-Hogg-Dube syndrome Am J Surg Pathol 2002 26 1542 1552 10.1097/00000478-200212000-00002 12459621

[B7] Khoo SK Bradley M Wong FK Hedblad MA Nordenskjold M Teh BT Birt-Hogg-Dube syndrome: mapping of a novel hereditary neoplasia gene to chromosome 17p12-q11.2 Oncogene 2001 20 5239 5242 10.1038/sj.onc.1204703 11526515

[B8] Schmidt LS Warren MB Nickerson ML Weirich G Matrosova V Toro JR Turner ML Duray P Merino M Hewitt S Pavlovich CP Glenn G Greenberg CR Linehan WM Zbar B Birt-Hogg-Dube syndrome, a genodermatosis associated with spontaneous pneumothorax and kidney neoplasia, maps to chromosome 17p11.2 Am J Hum Genet 2001 69 876 882 10.1086/323744 11533913 PMC1226073

[B9] Nickerson ML Warren MB Toro JR Matrosova V Glenn G Turner ML Duray P Merino M Choyke P Pavlovich CP Sharma N Walther M Munroe D Hill R Maher E Greenberg C Lerman MI Linehan WM Zbar B Schmidt LS Mutations in a novel gene lead to kidney tumors, lung wall defects, and benign tumors of the hair follicle in patients with the Birt-Hogg-Dube syndrome Cancer Cell 2002 2 157 164 10.1016/S1535-6108(02)00104-6 12204536

[B10] Khoo SK Giraud S Kahnoski K Chen J Motorna O Nickolov R Binet O Lambert D Friedel J Lévy R Ferlicot S Wolkenstein P Hammel P Bergerheim U Hedblad MA Bradley M Teh BT Nordenskjöld M Richard S Clinical and genetic studies of Birt-Hogg-Dube syndrome J Med Genet 2002 39 906 912 10.1136/jmg.39.12.906 12471204 PMC1757219

[B11] Leter EM Koopmans AK Gille JJ van OSTA Vittoz GG David EF Jaspars EH Postmus PE van Moorselaar RJ Craanen ME Starink TM Menko FH Birt-Hogg-Dube syndrome: clinical and genetic studies of 20 families J Invest Dermatol 2008 128 45 49 10.1038/sj.jid.5700959 17611575

[B12] Vocke CD Yang Y Pavlovich CP Schmidt LS Nickerson ML Torres-Cabala CA Merino MJ Walther MM Zbar B Linehan WM High frequency of somatic frameshift *BHD *gene mutations in Birt-Hogg-Dube-associated renal tumors J Natl Cancer Inst 2005 97 931 935 10.1093/jnci/dji154 15956655

[B13] Okimoto K Sakurai J Kobayashi T Mitani H Hirayama Y Nickerson ML Warren MB Zbar B Schmidt LS Hino O A germ-line insertion in the Birt-Hogg-Dube (*BHD*) gene gives rise to the Nihon rat model of inherited renal cancer Proc Natl Acad Sci USA 2004 101 2023 2027 10.1073/pnas.0308071100 14769940 PMC357045

[B14] Lingaas F Comstock KE Kirkness EF Sorensen A Aarskaug T Hitte C Nickerson ML Moe L Schmidt LS Thomas R Breen M Galibert F Zbar B Ostrander EA A mutation in the canine BHD gene is associated with hereditary multifocal renal cystadenocarcinoma and nodular dermatofibrosis in the German Shepherd dog Hum Mol Genet 2003 12 3043 3053 10.1093/hmg/ddg336 14532326

[B15] Baba M Furihata M Hong SB Tessarollo L Haines DC Southon E Patel V Igarashi P Alvord WG Leighty R Yao M Bernardo M Ileva L Choyke P Warren MB Zbar B Linehan WM Schmidt LS Kidney-targeted Birt-Hogg-Dube gene inactivation in a mouse model: Erk1/2 and Akt-mTOR activation, cell hyperproliferation, and polycystic kidneys J Natl Cancer Inst 2008 100 140 154 10.1093/jnci/djm288 18182616 PMC2704336

[B16] Chen J Futami K Petillo D Peng J Wang P Knol J Li Y Khoo SK Huang D Qian CN Zhao P Dykema K Zhang R Cao B Yang XJ Furge K Williams BO Teh BT Deficiency of FLCN in mouse kidney led to development of polycystic kidneys and renal neoplasia PLoS One 2008 3 e3581 10.1371/journal.pone.0003581 18974783 PMC2570491

[B17] Hasumi Y Baba M Ajima R Hasumi H Valera VA Klein ME Haines DC Merino MJ Hong SB Yamaguchi TP Schmidt LS Linehan WM Homozygous loss of BHD causes early embryonic lethality and kidney tumor development with activation of mTORC1 and mTORC2 Proc Natl Acad Sci USA 2009 106 18722 18727 10.1073/pnas.0908853106 19850877 PMC2765925

[B18] Hartman TR Nicolas E Klein-Szanto A Al Saleem T Cash TP Simon MC Henske EP The role of the Birt-Hogg-Dube protein in mTOR activation and renal tumorigenesis Oncogene 2009 28 1594 1604 10.1038/onc.2009.14 19234517 PMC2664853

[B19] Hudon V Sabourin S Dydensborg AB Kottis V Ghazi A Paquet M Crosby K Pomerleau V Uetani N Pause A Renal tumor suppressor function of the Birt-Hogg-Dube syndrome gene product folliculin J Med Genet 2010 47 182 189 10.1136/jmg.2009.072009 19843504

[B20] Baba M Hong SB Sharma N Warren MB Nickerson ML Iwamatsu A Esposito D Gillette WK Hopkins RF Hartley JL Furihata M Oishi S Zhen W Burke TR Jr Linehan WM Schmidt LS Zbar B Folliculin encoded by the BHD gene interacts with a binding protein, FNIP1, and AMPK, and is involved in AMPK and mTOR signaling Proc Natl Acad Sci USA 2006 103 15552 15557 10.1073/pnas.0603781103 17028174 PMC1592464

[B21] Hasumi H Baba M Hong SB Hasumi Y Huang Y Yao M Valera VA Linehan WM Schmidt LS Identification and characterization of a novel folliculin-interacting protein FNIP2 Gene 2008 415 60 67 10.1016/j.gene.2008.02.022 18403135 PMC2727720

[B22] van Slegtenhorst M Khabibullin D Hartman TR Nicolas E Kruger WD Henske EP The Birt-Hogg-Dube and tuberous sclerosis complex homologs have opposing roles in amino acid homeostasis in *Schizosaccharomyces pombe* J Biol Chem 2007 282 24583 24590 10.1074/jbc.M700857200 17556368

[B23] Yang Y Padilla-Nash HM Vira MA Abu-Asab MS Val D Worrell R Tsokos M Merino MJ Pavlovich CP Ried T Linehan WM Vocke CD The UOK 257 cell line: a novel model for studies of the human Birt-Hogg-Dube gene pathway Cancer Genet Cytogenet 2008 180 100 109 10.1016/j.cancergencyto.2007.10.010 18206534 PMC2440670

[B24] Singh SR Zhen W Zheng Z Wang H Oh SW Liu W Zbar B Schmidt LS Hou SX The *Drosophila *homolog of the human tumor suppressor gene *BHD *interacts with the JAK-STAT and Dpp signaling pathways in regulating male germline stem cell maintenance Oncogene 2006 25 5933 5941 10.1038/sj.onc.1209593 16636660

[B25] Derynck R Feng XH TGF-beta receptor signaling Biochim Biophys Acta 1999 1333 F105 150 10.1016/s0304-419x(97)00017-6 9395284

[B26] Shi Y Massague J Mechanisms of TGF-beta signaling from cell membrane to the nucleus Cell 2003 113 685 700 10.1016/S0092-8674(03)00432-X 12809600

[B27] de Caestecker MP Piek E Roberts AB Role of transforming growth factor-beta signaling in cancer J Natl Cancer Inst 2000 92 1388 1402 10.1093/jnci/92.17.1388 10974075

[B28] Markowitz S Wang J Myeroff L Parsons R Sin L Lutterbaugh J Inactivation of the type II TGF-beta receptor in colon cancer cells with microsatellite instability Science 1995 268 1336 1338 10.1126/science.7761852 7761852

[B29] Parsons R Myeroff LL Liu B Willson JK Markowitz SD Kinzler KW Vogelstein B Microsatellite instability and mutations of the transforming growth factor beta type II receptor gene in colorectal cancer Cancer Res 1995 55 5548 5550 7585632

[B30] Lu SL Akiyama Y Nagasaki H Saitoh K Yuasa Y Mutations of the transforming growth factor-beta type II receptor gene and genomic instability in hereditary nonpolyposis colorectal cancer Biochem Biophys Res Commun 1995 216 452 457 10.1006/bbrc.1995.2644 7488133

[B31] Hahn SA Schutte M Hoque AT Moskaluk CA da Costa LT Rozenblum E Weinstein CL Fischer A Yeo CJ Hruban RH Kern SE DPC4, a candidate tumor suppressor gene at human chromosome 18q21.1 Science 1996 271 350 353 10.1126/science.271.5247.350 8553070

[B32] MacGrogan D Pegram M Slamon D Bookstein R Comparative mutational analysis of DPC4 (Smad4) in prostatic and colorectal carcinomas Oncogene 1997 15 1111 1114 10.1038/sj.onc.1201232 9285566

[B33] Han SU Kim HT Seong DH Loss of the Smad3 expression increases susceptibility to tumorigenicity in human gastric cancer Oncogene 2004 23 1333 1341 10.1038/sj.onc.1207259 14647420

[B34] Takagi Y Kohmura H Futamura M Kida H Tanemura H Shimokawa K Saji S Somatic alterations of the DPC4 gene in human colorectal cancers in vivo Gastroenterology 1996 111 1369 1372 10.1053/gast.1996.v111.pm8898652 8898652

[B35] Chen YG Wang Q Lin SL Chang CD Chuang J Ying SY Activin signaling and its role in regulation of cell proliferation, apoptosis, and carcinogenesis Exp Biol Med (Maywood.) 2006 231 534 544 16636301 10.1177/153537020623100507

[B36] Maeshima A Miya M Mishima K Yamashita S Kojima I Nojima Y Activin A: autocrine regulator of kidney development and repair Endocr J 2008 55 1 9 10.1507/endocrj.KR-113 17827789

[B37] Ioachim E Michael MC Salmas M Damala K Tsanou E Michael MM Malamou-Mitsi V Stavropoulos NE Thrombospondin-1 expression in urothelial carcinoma: prognostic significance and association with p53 alterations, tumour angiogenesis and extracellular matrix components BMC Cancer 2006 6 140 10.1186/1471-2407-6-140 16732887 PMC1538616

[B38] Sutton CD O'Byrne K Goddard JC Marshall LJ Jones L Garcea G Dennison AR Poston G Lloyd DM Berry DP Expression of thrombospondin-1 in resected colorectal liver metastases predicts poor prognosis Clin Cancer Res 2005 11 6567 6573 10.1158/1078-0432.CCR-05-0439 16166434

[B39] Rice AJ Steward MA Quinn CM Thrombospondin 1 protein expression relates to good prognostic indices in ductal carcinoma in situ of the breast J Clin Pathol 2002 55 921 925 10.1136/jcp.55.12.921 12461058 PMC1769827

[B40] Guerrero D Guarch R Ojer A Casas JM Ropero S Mancha A Pesce C Lloveras B Garcia-Bragado F Puras A Hypermethylation of the thrombospondin-1 gene is associated with poor prognosis in penile squamous cell carcinoma BJU Int 2008 102 747 755 10.1111/j.1464-410X.2008.07603.x 18336597

[B41] Rozen S Skaletsky H Primer3 on the www for general users and for biologist programmers Methods Mol Biol 2000 132 365 386 10547847 10.1385/1-59259-192-2:365

