# Supplementary material for: Tumor suppressor FLCN inhibits tumorigenesis of a FLCN-null renal cancer cell line and regulates expression of key molecules in TGF-β signaling
Source: Mol Cancer. 2010 Jun 23;9:160. doi: 10.1186/1476-4598-9-160 (PMC2907329; doi:10.1186/1476-4598-9-160)
Supplement: Additional file 1 — table S1, and figures S1, S2, S3. Table S1. Frequency and characteristics of tumors that developed from different UOK257 cell lines in athymic nude mice. Figure S1. PCR amplification of endogenous FLCN (endo) and FLCN transgene (tg) from the xenograft tumors. Figure S2. Deregulation of the key molecules in TGF-β signaling by FLCN expression. Figure S3. Quantitative RT-PCR for TGFB2, INHBA, THBS1, GREM1 and SMAD3 in the UOK257 cell lines expressing either mutant or wild-type FLCN. [file 1476-4598-9-160-S1.PDF]

Table S1. Frequency and characteristics of tumors that developed from different UOK257 cell lines in athymic nude mice.

**UOK257**	**Solid tumors** (incidence)	**Volume** (mm^3)	**Histology**	***Tumor patch** (incidence)	**Histology**	**Avg. Days**
P	19/21	1732±11782	Clear cell, high grade	2/21	Clear cell, high grade	310
H255R	8/8	1275±773	Clear cell, high grade	0/8	-	280
3	2/10	624±516	Clear cell, high grade	5/10	Clear cell, papillary, low to high grade	235
4	0/10	-	-	4/10	Clear cell, low grade	297
2	0/17	-	-	2/17	Clear cell, low grqade	335
6	0/8	-	-	0/8	-	327

NOTE: P, parental; H255R, mutant FLCN-H255R; 2, 3, 4, and 6, wild-type *FLCN* expressing UOK257 lines. *Tumor cells grown as a patch of <1 mm thickness.

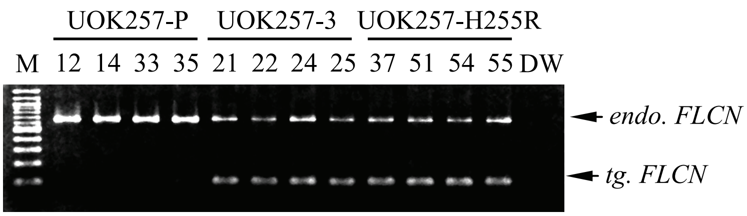

Figure S1. PCR amplification of endogenous *FLCN* (endo) and *FLCN* transgene (tg) from the tumors. Numbers, mouse number; M, size marker; DW, distilled water control.

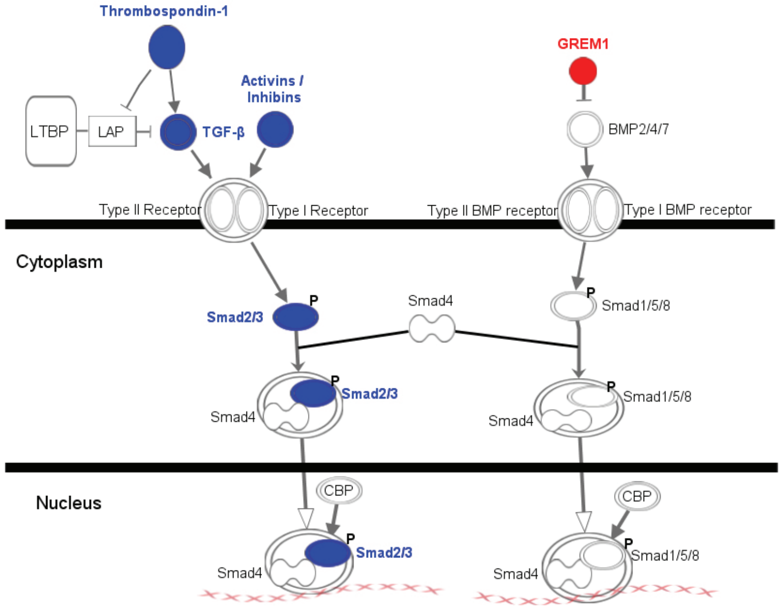

Figure S2. Deregulation of the key molecules in TGF-β signaling by *FLCN* expression

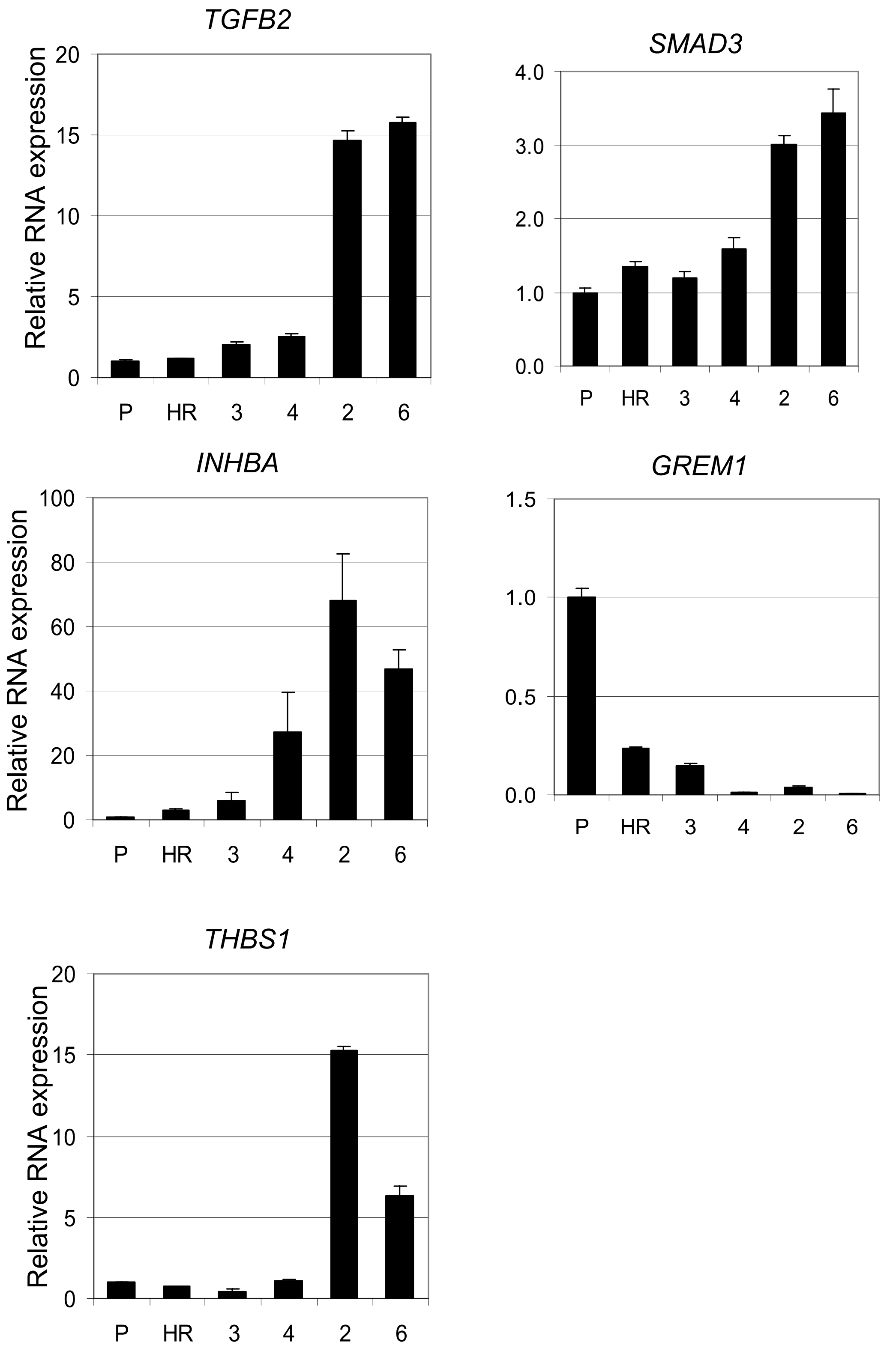

Figure S3. Quantitative RT-PCR for *TGFB2*, *INHBA*, *THBS1*, *GREM1* and *SMAD3* in the UOK257 cell lines expressing either mutant or wild-type *FLCN*. Columns, mean; bars, +SD (n=3).
